# Using augmented reality to enhance wax up training in dental education a feasibility study

**DOI:** 10.1038/s41598-025-10474-4

**Published:** 2025-07-29

**Authors:** Johannes Schrenker, Michael del Hougne, Marc Schmitter, Christian Höhne

**Affiliations:** Department of Prosthodontics, University of Würzburg, Pleicherwall 2, 97070 Würzburg, Germany

**Keywords:** Dental education, Wax-up, Augmented reality, AR, Smartphone application, App, Dentistry, Dental education

## Abstract

Wax-up practice is an integral part of dental education, teaching students the morphology of teeth and correct handling of waxing tools. Currently wax-ups need to be presented and evaluated by a dentist involved in dental education. The aim of this study was the development of a smartphone application utilizing augmented reality to improve self-evaluation and learning effects of beginners doing wax-up practice. The application was developed for devices running the iOS operating-system using the Swift language. The Swift-libraries UIKit, ArKit and SceneKit were used. The application was test-run on an iPhone 14 Pro, using an iOS developer account. An integral component of the application is the use of 3D-printed wax-up templates, which were manufactured with FDM printers. The resulting smartphone application enables dental students and teaching dentists to inspect the wax-up template using the device’s camera to view the optimal wax-up overlaying the template. Different cusps can be displayed, hidden or altered in their transparency to further improve feedback on the students’ practice. The aim of creating a supportive application for wax-up practice as a proof of concept was fulfilled and even exceeded the initial expectations regarding the accuracy and consistency of object tracking in the augmented reality. The acceptance amongst students and teaching staff, as well as a comparison against traditional methods of teaching wax-up remain to be evaluated.

## Introduction

**Fig. 1:**
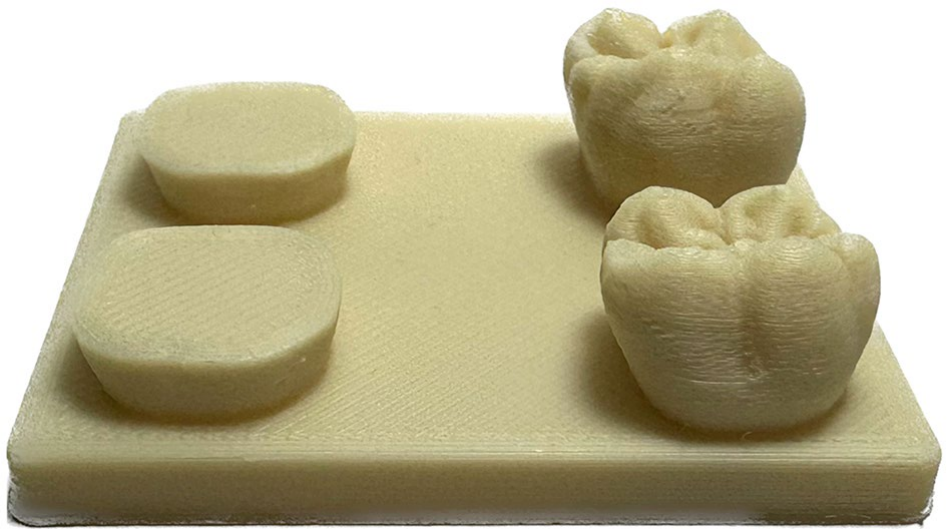
FDM 3D printed wax-up template with one upper and one lower jaw molar and according dies.

Augmented reality is defined as a combination of virtual objects with the real world. This requires a device offering an array of inputs; a camera to capture the physical world, additional trackers and sensors, sufficient computing power and a visual output for the user, mostly a display, as summarized by Sünger and Çankaya^1^. Smartphones and comparable devices provide all these critical components and are readily available nowadays. They offer the perfect platform for leveraging augmented reality in dental education. In fall 2021 a new curriculum was established in the education of dental students in Germany. This resulted in a substantial change, as the practical course in the first term of dental undergraduate education is shortened. In prior regulations this course took up the entire first semester. This has now been reduced to 42 h of practical work. Subjects of this new course at our university are wax-up practice, preparation for crowns and the production of removable prosthetic dentures. Only two days are scheduled for wax-up practice. The aim of the wax-up practice is the reproduction of an upper and lower jaw molar on corresponding templates. The cusps have to be waxed in different colors according to a master template as demonstrated by supervising dentists. Students are required to present each step individually to their supervisors. The Department of Prosthodontics introduced the production of the templates for wax-up practices with a 3D FDM printer (Fig. 1), enabling a time saving workflow and relieving the students, as the templates had to be cast in gypsum by the students themselves before. As practicing the handling of gypsum is scheduled for subsequent courses, focus on wax-up practice is enhanced.

For accelerated learning outcomes in this shortened timeframe, the idea of a smartphone application was conceptualized, aiding the tutoring of wax-up practices, and alleviating the bottleneck of two supervising dentists responsible for various students simultaneously. The application developed modernizes education, digitalizes the preclinical stages of dentistry and ensures a foundation for further, digital explorations, which will most likely be the future of dental education^2^. To the authors’ knowledge, there have just been studies conducted using AR (augmented reality) and VR (virtual reality) to visualize teeth and simulate procedures, however, no use of AR combined with real teaching equipment for further enhancement of the capabilities of wax-up-training has been covered yet. There has been a vast amount of standalone AR and VR projects already, emphasizing a virtual learning experience^4,9^ or a simulation on different treatments^3,6,10^. All systematic reviews attribute AR and WR a growing importance as a future teaching-method^5,7,8^.

## Materials and methods

### FDM-Printed wax-up-templates

Students are handed pre-produced 3D printed wax-up templates, based on the gypsum template utilized previously. The template’s model was prepared for 3D printing with Ultimakers Cura (Version 5.3.1, UltiMaker, Utrecht, Netherlands), a 3D-slicing-software. These templates are manufactured by several modified FDM-printers (Anycubic Kobra, Anycubic, Shenzhen, China). The material for production is a PLA-filament - an industry standard. The material of production is a high-quality, food-safe, biocompatible, sterilizable and temperature resistant material (Green-TEC PRO Natur, Extrudr, Lustenau, Austria). It is made completely from renewable raw materials and biodegradable according to DIN EN ISO 14,855. The use of FDM printed wax-up templates is an integral part of the app-development, as all students are provided a unified and identical model for practice. Due to the digital creation of the template, an accurate and detailed 3D model is available for further utilization in the application.

### Basics of programming

The application was programmed for devices running the iOS operating system and test run on an iPhone 14 Pro to make use of the integrated LiDar-sensor for additional spatial awareness. This device also features an array of three physical lenses, a wide angle lens (13 mm, f2.2), a telephoto lens (77 mm, f 2.8) and a main 48 MP lens (24 mm, f 1.78) with a minimum focus distance of approximately 20 cm. To account for lens distortion - and therefore properly correct the AR-overlay - only the main lens is eligible. Due to framework dependency the minimum requirement of iOS is version 16.2 (currently 16.5). Development versions of the application were installed on the devices and maintained with an Apple developer account. The application was programmed using the Swift language (version 5.5) in Xcode (version 14.3–14E222b) on MacOS Ventura (version 3.3.1 (a)). A combination of pure coding and visual scripting using Xcode’s storyboard feature was utilized during development.

Multiple Swift frameworks were integrated to enable certain features:

UIKit / CocoaTouch: UIKit is used to design and operate a graphical user interface, stylistically oriented along native iOS design components.

ARKit: The ARKit framework is used to handle a device’s camera input and calculate spatial orientation, as well as starting and maintaining AR sessions in the viewcontroller class.

SceneKit: SceneKit is the underlying 3D rendering engine for creating, manipulating and properly integrating the overlays into the running AR session.

### Management of 3D-models and storage of assets

The stereolithography-fileformat (.stl) was chosen to correctly import the digital model of the wax-up template into the Cura software and translate it into 3D printer instructions. Blender (version 3.5.1), a free and opensource 3D modelling and model editing software, was utilized for creating the model assets for the AR overlay in the correct dimensions, as well as converting all 3D assets used into the Collada fileformat (.dae). Upon import into Xcode, the Collada files were converted into SceneKit scenes (.scn). Materials were applied by code in a later step. Images embedded in the application can be separated into two groups: first, the images that serve an aesthetic purpose, such as the launch screen or UI components. These were imported to the asset folder in the portable network graphics file format (.png). Second, the images used as tracking markers. These were imported into an AR resource group as an AR reference image - an Xcode specific file format that allows the definition of fixed physical sizes for the target image.

### Approaching development with instructor feedback

A direct and flexible development of the application was enabled, as programming was conducted by a teaching faculty dentist, resulting in an elimination of communication hassle and waiting times. During development, the application was presented to dentists experienced in teaching wax-up-practice in preclinical and undergraduate students. Feedback regarding functionality and design was implemented and the application was reevaluated. This procedure was repeated over multiple iterations to achieve the final stage.

## Results

### Tracking methods

**Fig. 2:**
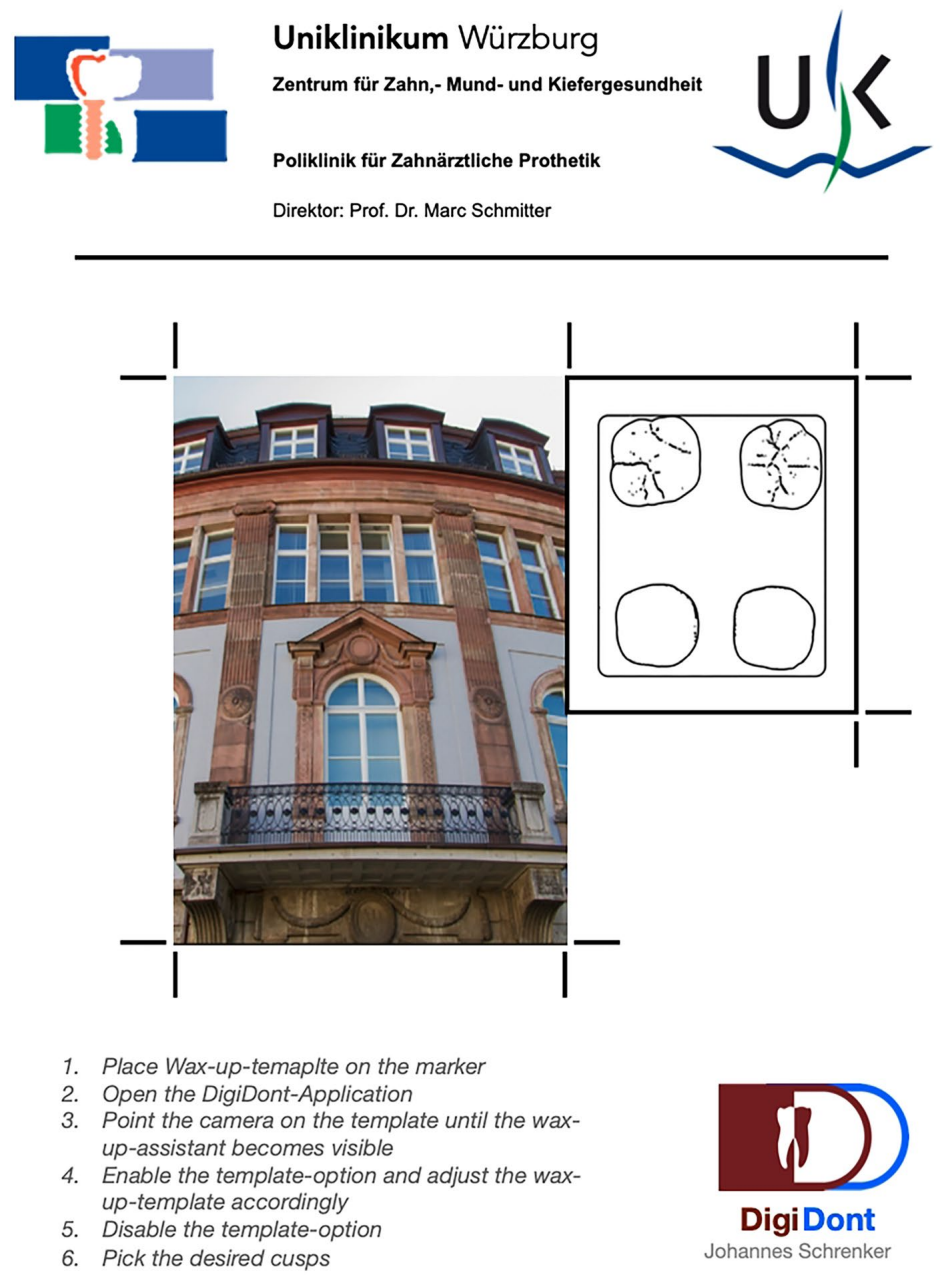
The tracking platform as a printout with instructions, a positioning field and a tracking marker.

During development of the application many hurdles had to be overcome. The most important problem to solve was the establishment of a fast, accurate and reliable object tracking method. Two methods of tracking were trialed against each other. The first method uses the recognition and spatial tracking of actual 3D objects by comparing them to 3D scanned versions of themselves. In this case, the object to be tracked was the wax-up template. In contrast to bigger objects, tracking of small objects such as the template proved to be unreliable and more importantly very inaccurate, often shifting the overlay by multiple millimeters. The second method utilizes image tracking instead of object tracking. In order to use this approach, a custom reusable tracking platform (Fig. 2) was constructed, which consists out of instructions for use, the tracking image itself and a placement marker for the wax-up template. As the tracked image and its physical size are known to the application, the spatial orientation of the phone and the cameras perspective can be calculated and then applied to the 3D overlay. This approach allows for a fluent and accurate tracking of the template, requiring the tracked image to be positioned on a flat surface and the tracked image to be always at least partially visible to the camera.

### Application optimizations and UI changes during development

**Fig. 3:**
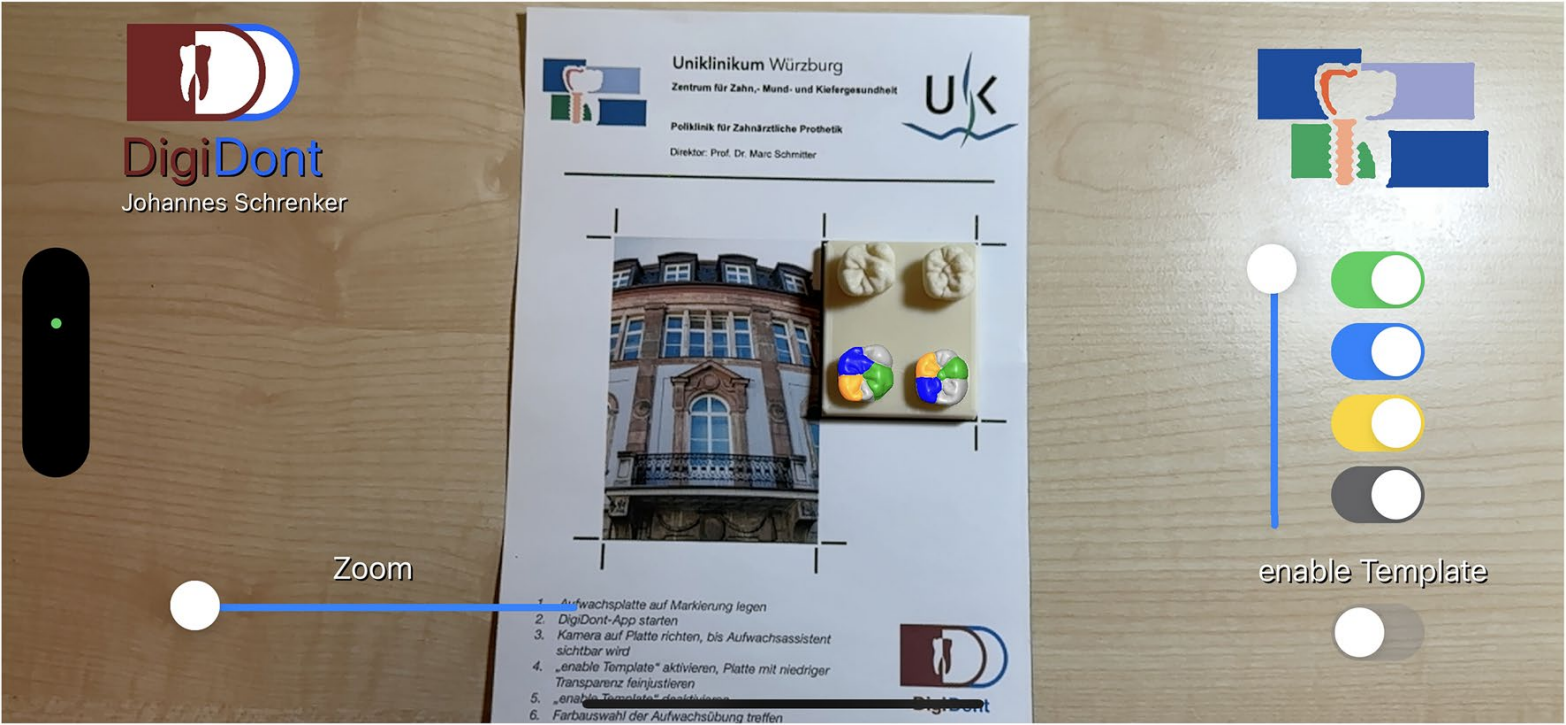
The application with a fully projected augmented reality overlay in a top-down view.

**Fig. 4:**
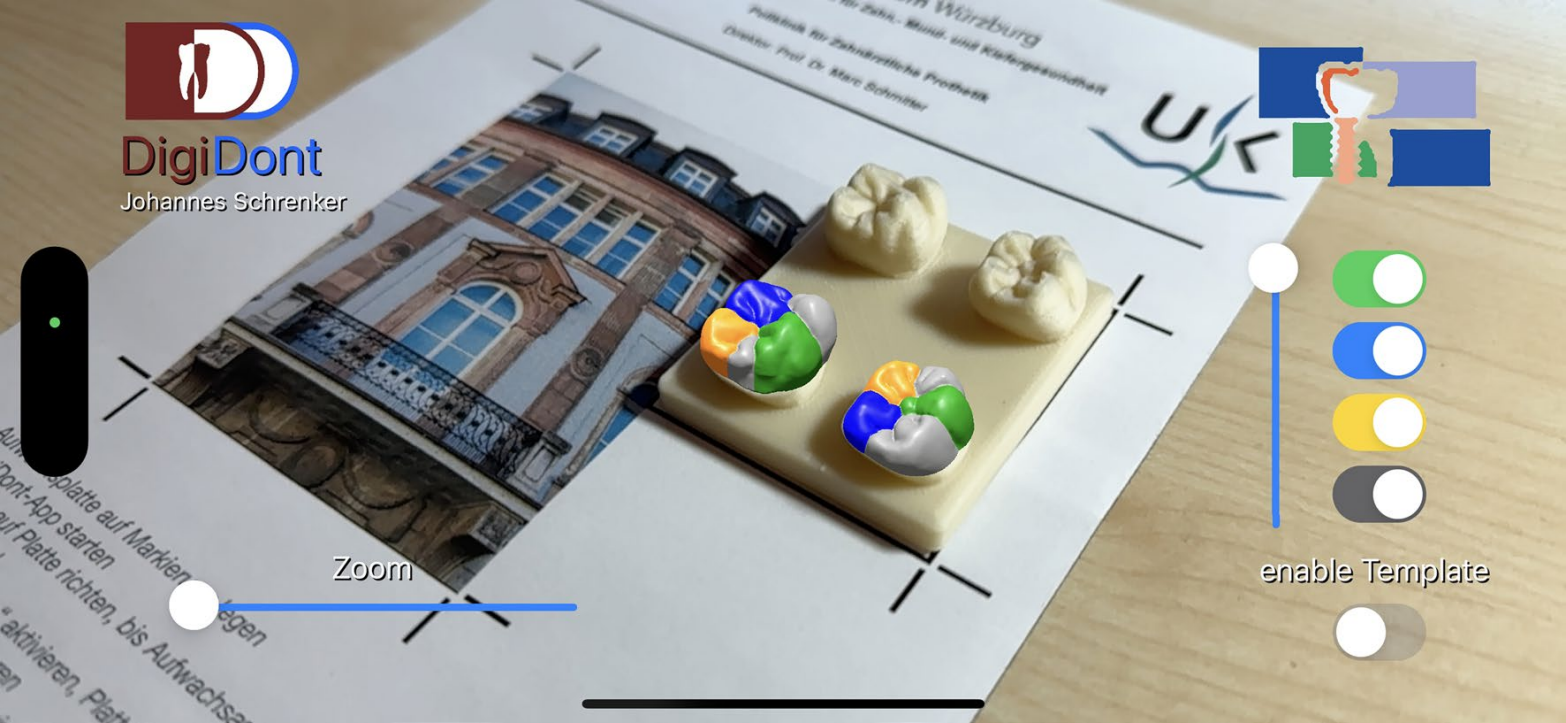
The application with a fully projected augmented reality overlay in a side view.

**Fig. 5:**
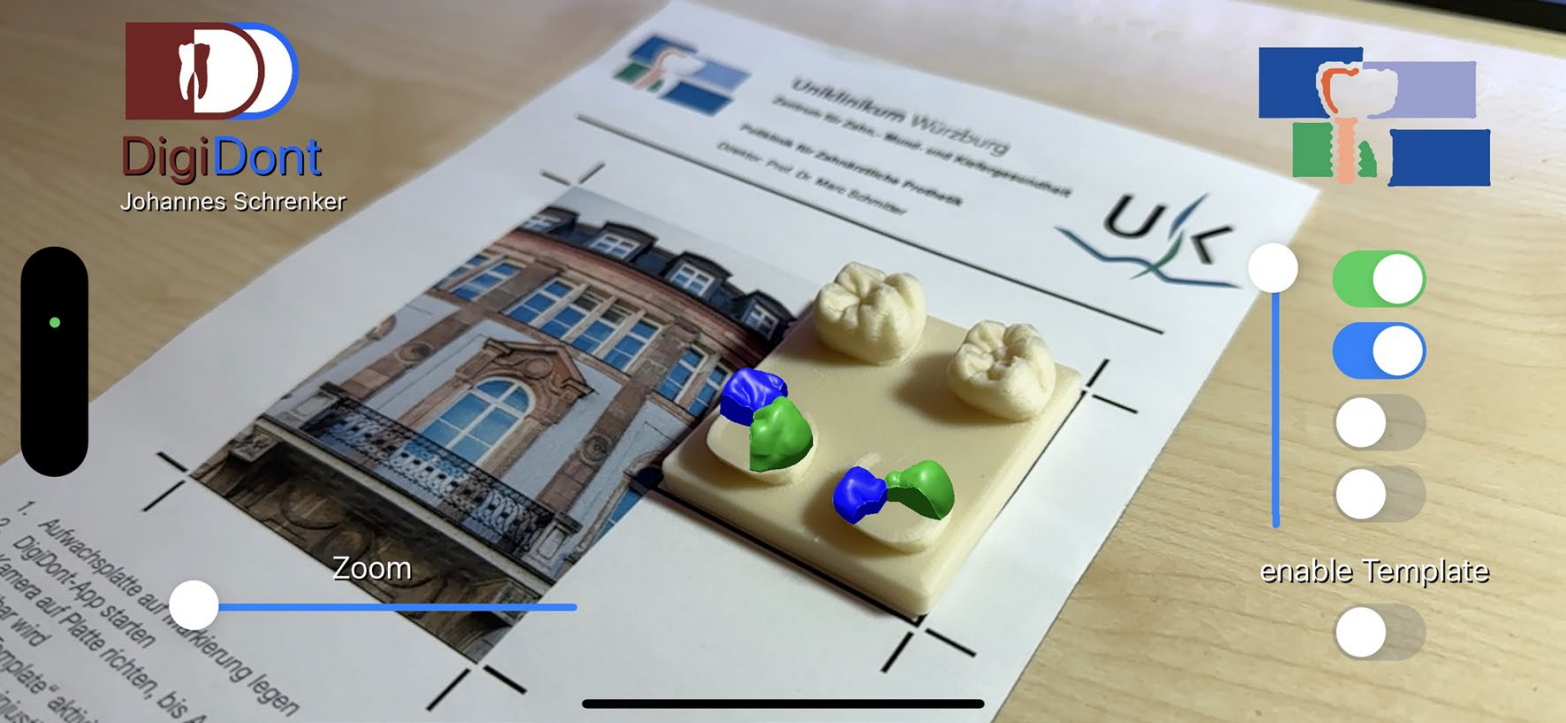
The application with a partially enabled projected augmented reality overlay in a side view.

**Fig. 6:**
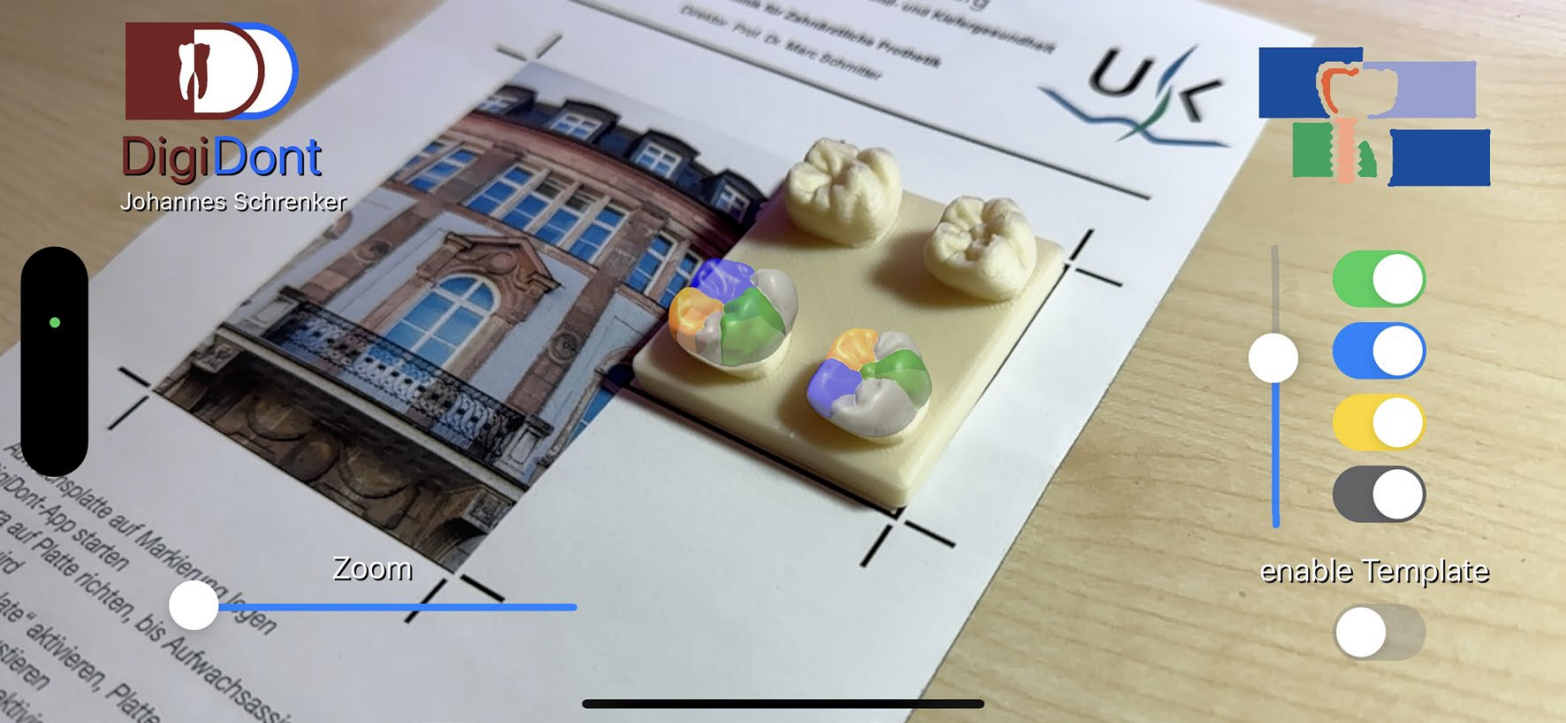
The application with a fully projected augmented reality overlay at reduced transparency in a side view.

**Fig. 7:**
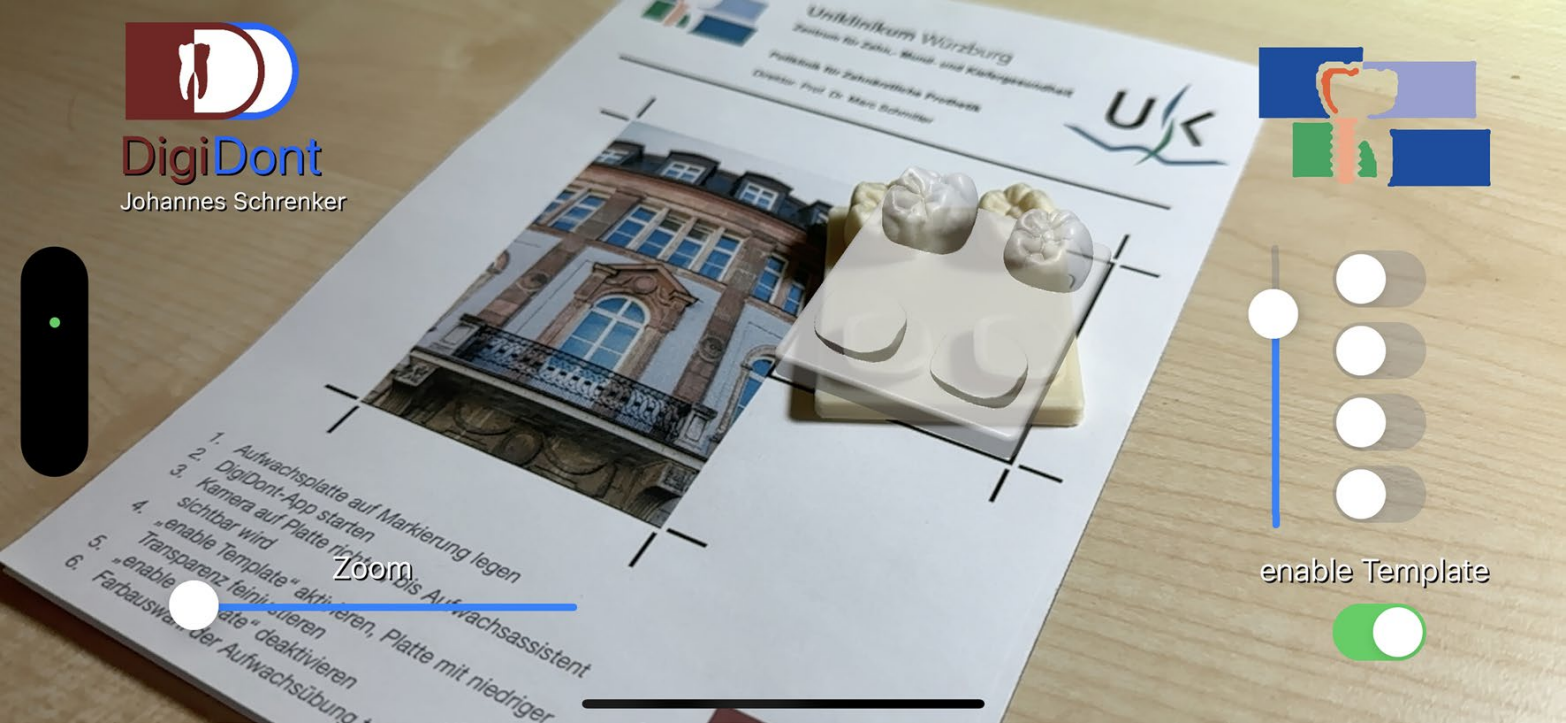
The expected position of the template is projected at reduced transparency.

After the ideal tracking method, utilizing the tracking markers, was determined, further optimizations had to embedded to prevent a jittering of the overlay. Changes included tweaking of the tracking algorithm and choosing a tracking image which is clearly distinguishable and rich in contrast and features. As a prove of concept, this first iteration of the app was presented to dentists involved in teaching preclinical and undergraduate students. Upon review some changes were suggested and implemented; the overlay, which was one model in a solid color previously was broken up into individual cusps and colored (Figs. 3 and 4) according to the physical wax-up template provided to the students. Furthermore, the ability to enable and disable the display of the individual cusps (Fig. 5) was implemented. In addition, a slider for changing the transparency (Fig. 6) of the entire overlay to improve the control of the wax-up-practice was added. Following another revision by the team of teaching dentists, several adaptions were embedded to the user interface as well as quality of life updates. As such, all important controls were moved to the right side of the screen, within the thumbs reach, allowing for one hand controls. An additional button was implemented to enable an overlay of the model of the wax-up template, which can be altered in transparency (Fig. 7). This step was taken to further improve the tracking accuracy, as the user is able to adjust the physical template to perfectly align with the virtually expected position. Finally, a zoom slider was added, circumventing two technical limitations of the framework and method of tracking. Given that the ARKit-Framework can solely use the iPhone’s main camera-lens for AR-Sessions, the user cannot move closer to the physical template than the camera’s minimum focus distance whilst maintaining a sharp image. The implementation of a digital zoom therefore enables the user a close-up look of the overlay and a sharp physical template. As the digital zoom only changes the content displayed on the screen and not the content seen by the device’s camera, the physical tracking marker can be outside of the bounds displayed on the screen.

### Final result and extent of features

**Fig. 8:**
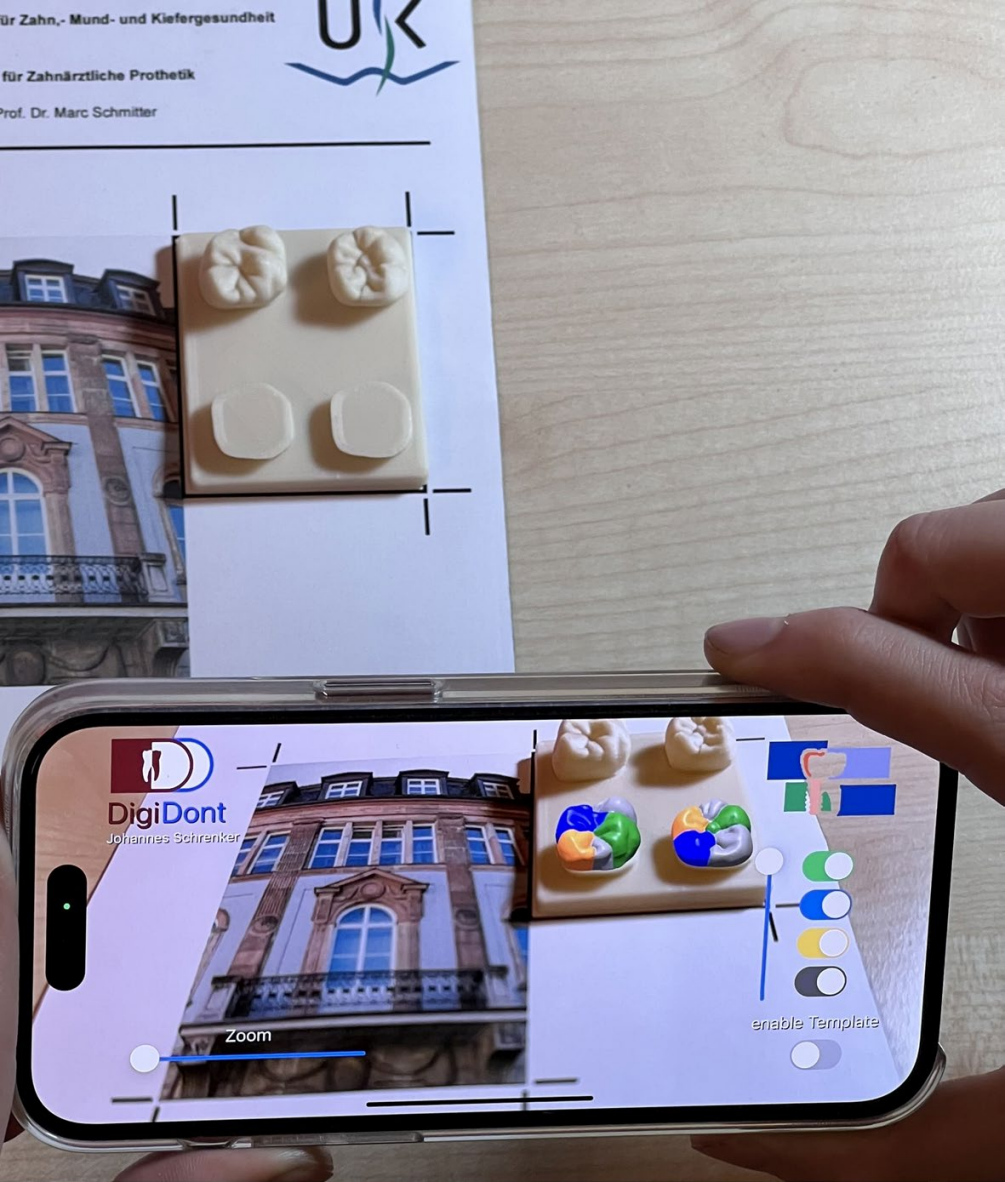
The view of a user running the application.

The final result (Fig. 8) is a lightweight and fast operating smartphone application running on iOS. The additional use of 3D printed wax-up templates, a custom designed tracking marker and software supported positioning allow for a reliable and accurate tracking of physical wax-up templates. The AR overlay of the cusp templates can be individually enabled or disabled and altered in transparency. Combined with the zoom feature the application is assumed to allow teaching dentists and preclinical students a faster and better control of their wax-up practice. Therefore, the aim of the study, to estimate whether the development of such an application is possible from a technical standpoint, was fulfilled.

The applications features include:

- Smooth and seamless AR-Tracking based on tracking-markers.
- Display of an AR-Overlay to help with wax-up-practice according to a master-template.
- Variable AR-Overlay with individually selectable cusps and transparency.
- Digital zoom for better detail-control without changing the camera-focus-length.

The application currently has to deal with following limitations:

- No AR-tracking without tracking-markers.
- Limited to iPhone 14, with further device-support planned.
- Limited to devices running the iOS operating-system.

## Discussion

### Practical acceptance and further testing

So far, the application was developed as a proof of concept for using augmented reality in dental education and for testing the various methods and accuracy of tracking. Even though this application revealed that these set out aims are achievable regarding the technical aspects, acceptance amongst students and teaching staff, as well as a comparison against traditional methods of teaching wax-up remain to be evaluated.

### Technical limitations

At the moment, the application is limited to run on the iPhone 14 Pro. Running the application on different devices would require a reorganization of the user interface, which can, however, be implemented easily. Regrettably, the application only can be run on devices within the iOS ecosystem, as the app is heavily based upon Swift dependent frameworks, so devices running Android likely will not ever be supported. Another technical limitation is that the app can currently only display an overlay of the optimal wax-up. The student or dental instructor still has to interpret the result and decide which cusps need further work. A fully automatic correction of the wax-up practice, with the application deciding autonomously on correct and incorrect parts, is not possible with the current level of smartphone technology. The built-in LIDAR sensor is by no means accurate enough to create a real-time 3D model of the waxed-up student practice for comparison with the expected outcome. A possible solution of this problem could be an artificial intelligence, trained on an appropriately sized set of pictures of acceptable wax-up practices, to then decide on the validity of the wax-up practice on a visual base, circumventing the translation of students’ practices into 3D models.

### Planned use

To ensure that every student, independent of the operating system of the personally owned device, is able to use the application during wax-up practice, providing iPads in the laboratory is planned, running the app together with the tracking platform. Those augmented reality stations are to be used in addition to the instructing dental teachers. To achieve this goal, support for more ios-devices will be added.

## Conclusion

The aim of this study was to explore the possibilities of using augmented reality in combination with physical teaching equipment in dental education. By developing a method of tracking the wax-up templates and designing a smartphone application with an appropriate user interface the aim of this study was fulfilled. However no test with students or teaching dentists were performed so far. Further practical trials and extensions to augmented reality in dental education are planned. The application provides a promising step into digitalization and modernization of preclinical education.

## Data Availability

The data that support the findings of this study are available from the corresponding author upon reasonable request.
